# Inactivation of Acetyl-CoA Acyltransferase 1 enhances the proliferation and motility of nasopharyngeal carcinoma cells

**DOI:** 10.1080/23723556.2025.2583342

**Published:** 2025-11-17

**Authors:** Wanqi Wei, Limei Li, Weilin Zhao, Shixing Zheng, Xiaoying Zhou, Haili Liang, Wen Wang, Feng He, Yushan Liang, Guangwu Huang, Zhe Zhang, Xue Xiao

**Affiliations:** a Department of Otolaryngology-Head and Neck Surgery, First Affiliated Hospital of Guangxi Medical University, Nanning, People's Republic of China; b Department of Pediatric Dentistry, College & Hospital of Stomatology, Guangxi Medical University, Nanning, People's Republic of China; c ENT Institute, Department of Otorhinolaryngology Fudan University, Shanghai, People's Republic of China; d Key Laboratory of High-Incidence-Tumor Prevention & Treatment (Guangxi Medical University), Ministry of Education, Nanning, People's Republic of China; e Guangxi Zhuang Autonomous Region Institute of Product Quality Inspection, Nanning, People's Republic of China

**Keywords:** Nasopharyngeal carcinoma, ACAA1, tumor suppressor, immune evasion, prognosis

## Abstract

Acetyl-CoA acyltransferase 1 (ACAA1), encoding the peroxisomal 3-ketoacyl-CoA thiolase (POT1), plays a pivotal role in the fatty acid beta-oxidation pathway. Accumulating evidence has linked this enzyme to the onset and development of diverse human malignancies. Here, we observed a marked downregulation of ACAA1 in nasopharyngeal carcinoma (NPC), which displayed an inverse correlation with the expression genes coded by Epstein-Barr virus (EBV). Receiver operating characteristic (ROC) curve and Kaplan-Meier survival analysis highlighted the potential of ACAA1 as a valuable diagnostic and prognostic biomarker for NPC. Next, gain-of- function experiments were conducted, and the results vividly illustrated that overexpression of ACAA1 potently impeded the proliferation, migration, and invasion of NPC cells. The inhibitory effect was further verified by the reduced Ki−67 staining intensity and the altered distribution pattern of actin filaments, which are crucial indicators of cell proliferation and motility. Gene ontology (GO) and Kyoto Encyclopedia of Genes and Genomes (KEGG) analyses revealed significant enrichment of immune-related pathways in NPC cells with elevated ACAA1 expression. Moreover, comprehensive xCell, ESTIMATE, and Immunophenoscore analyses underscored the positive association between ACAA1 and immune cell infiltration and the tumor immune effective microenvironment in NPC. Especially, a positive correlation between ACAA1 expression in tumor cells and six immune checkpoint-related genes, namely CD27, PDCD1, CD86, BTLA, TIGIT, and CD28, on immune cells within the tumor microenvironment. Collectively, our findings highlight the potential of ACAA1 as a tumor - suppressor gene and suggest its possible involvement in the immune evasion mechanisms of NPC. This study may provide novel insights into the molecular pathogenesis of NPC and offer new therapeutic targets for this malignancy.

## Introduction

Nasopharyngeal carcinoma (NPC) is a malignant tumour originating from the nasopharyngeal epithelium, with a pronounced geographical and ethnic predisposition.^1^ Although rare globally, NPC exhibit a particularly high incidence in southern China where annual rates can exceed 25 cases per 100,000 individuals.^2^ Its aetiology involves a complex interplay of Epstein-Barr virus (EBV) infection, environmental carcinogens, and genetic susceptibility^3^. Although radiotherapy-based regimens have improved outcomes, many patients still experience local recurrence, distant metastasis, and therapeutic resistance, underscoring the need for deeper molecular insights and more effective therapeutic targets.^4^^,^^5^

Metabolic reprogramming is a recognised hallmark of cancer, with aberrant fatty acid metabolism increasingly implicated in tumour progression and survival.^6^^,^^7^ In NPC, key enzymes in lipid metabolism—such as carnitine palmitoyl transferase 1 (CPT1A), sterol regulatory element-binding protein 1 (SREBP1), and adipose triglycerol lipase (ATGL)—have been shown to contribute to malignant phenotypes.^7–9^ Acetyl-CoA acyltransferase 1 (ACAA1), which catalyses the final step in peroxisomal *β*-oxidation of very long-chain fatty acids, plays a crucial role in maintaining lipid homoeostasis.^10^^,^^11^ However, its expression and functional significance in cancer remain ambiguous and context-dependent. For instance, ACAA1 is upregulated in the luminal androgen receptor subtype of triple-negative breast cancer, where its inhibition attenuates proliferation and synergises with CDK4/6 inhibitors.^12^ In contrast, ACAA1 is frequently downregulated in other malignancies, including gastric, lung, colorectal, thyroid, and renal clear cell carcinomas.^13–17^ Despite these associations, the functional role of ACAA1 in NPC, and its broader implications in tumour immunity, remains largely unexplored.

Emerging evidence indicates that lipid metabolic reprogramming not only supports tumour growth but also dynamically shapes the immune landscape of the tumour microenvironment (TME).^18^^,^^19^ The TME is a multicellular ecosystem wherein metabolic crosstalk between tumour and immune cells can either promote or suppress antitumor responses.^20^^,^^21^ Immune evasion, mediated through such metabolic interactions, represents a critical barrier to therapy.^22^ Interestingly, ACAA1 has been implicated in immune regulation. Its expression inversely correlates with T cell and neutrophil infiltration in lung adenocarcinoma, suggesting a potential role in modulating immune cell activity.^23^^,^^24^ These observations position ACAA1 at the intersection of lipid metabolism and immune regulation, raising compelling questions about its role in immune-evasive niches such as those in NPC.

Motivated by our previous bioinformatic analysis revealing significant downregulation of ACAA1 in NPC,^25^ we designed this study to systematically evaluate its clinical relevance, biological functions, and immunomodulatory potential. We aim to validate ACAA1 expression patterns in NPC clinical specimens and assess their prognostic significance; investigate its role in NPC progression through functional assays; and elucidate how ACAA1 modulates the immune landscape of NPC. By integrating mechanistic studies with immune correlate analyses, this work seeks to provide foundational insights into ACAA1 as a metabolic immune modulator and its potential as a biomarker or therapeutic target in NPC.

## Materials and methods

### Ethics statement

This study was granted approval by the Ethics Committee of the First Affiliated Hospital of Guangxi Medical University (2022-KT−243). Comprehensive information regarding the study's objectives, methodologies, and potential risks was disclosed to all participants, and written consents were duly obtained from each donor.

### Cell lines and human tissues

Five NPC cell lines (CNE1,TW03,5-8F, HONE1, and HK1) were maintained in Dulbecco’s modified medium (DMEM, Life Technologies, Gibco, C11995500BT) with 10% foetal bovine serum (FBS; Life Technologies, Gibco, 10091148) at 37℃ in a 5% CO_2_ atmosphere. The non-malignant nasopharyngeal epithelial cell line (NP460) was cultured in defined keratinocyte-serum free medium (KSFM, Life Technologies, Gibco, 10744019).

Nineteen newly diagnosed primary NPC biopsies were collected from the Department of Otolaryngology-Head and Neck Surgery, First Affiliated Hospital of Guangxi Medical University (Nanning, China). Diagnosis was confirmed by pathologists according to the World Health Organisation classification. Eighteen normal nasopharyngeal epithelium (NNE) tissue samples with chronic inflammation were included as controls. RNA was extracted from 18 NNE and 19 NPC samples.

A tissue microarray containing 125 NPC tissue samples was purchased from Shanghai Outdo Biotech Co., Ltd. (Shanghai, China; Cat No: HNasN129Su01). Fourteen normal nasopharyngeal epithelium (NNE) tissue samples were included as controls.

### Plasmids, reagents, and antibodies

ACAA1-overexpressing plasmids (pCMV6-Entry-ACAA1) and control plasmid (pCMV6-Entry) were purchased (Origene, PS100001). Transfection was performed using Lipofectamine 3000 (Life Technologies, L3000015), according to the manufacturer's protocol. The following antibodies and fluorescent dyes were used: ACAA1 (Abcam, ab90647), GAPDH (Proteintech, HRP60004), anti-rabbit IgG,HRP-linked antibody (Cell Signalling, 7074), anti-mouse IgG, HRP-linked antibody (Cell Signalling, 7076), Ki67 (Cell Signalling, 9449), phalloidin (Invitrogen, A12381) and DAPI (Solarbio, C0065).

### Raw data acquisition

Microarray data from datasets GSE12452, GSE180272, GSE53819, GSE61218, GSE64634, and GSE102349 were downloaded from the GEO database (http://www.ncbi.nlm.nih.gov/geo/). GSE12452 and GSE64634 were based on the GPL570 platform; GSE180272 on the GPL16956 platform; GSE53819 on the GPL6480 platform; and GSE61218 on the GPL19061 platform. These datasets were used to confirm ACAA1 expression in NPC. GSE102349, based on GPL11154, was used for subsequent analysis of ACAA1 expression correlations with tumour stage, EBV-encoded genes, TME subtypes, survival, and immune-related indexes.

### Transcriptional analysis of ACAA1

Quantitative real-time polymerase chain reaction (qPCR) was performed to validate ACAA1 expression in NPC cell lines and primary NPC tissues, using NP460 and NNE tissues as controls. Total RNA was extracted using TRIzol reagent (Life Technologies, Invitrogen, USA).^26^ First-strand cDNA synthesis was performed using RevertAid First Strand cDNA Synthesis Kit (Life Technologies, Invitrogen, USA), and qPCR was carried out with PowerUp SYBR Green PCR Master Mix (Applied Biosystems, USA). *GAPDH* was used as an internal control. The primer sequences were as follows:

*ACAA1*-Forward: 5’-CATCTGTGTCGGAAATGTGC−3’,

*ACAA1*-Reverse: 5’-TTCTGATGCCACCTGCTATG−3’,

*GAPDH*-Forward: 5’-AAGCTCACTGGCATGGCCTT−3’,

*GAPDH*-Reverse: 5’-CTCTCTTCCTCTTGTGCTCTTG−3’.

The PCR conditions: 95℃ for 30 s, then 40 cycles at 95℃ for 5 s, 60℃ for 30 s. Relative *ACAA1* expression was determined using the 2^−△△Ct^ method, and reactions were performed in triplicate.

### Western blot analysis

Protein was extracted using RIPA lysis buffer (Solarbio, R0010), separated by 10% SDS-PAGE and transferred to nitrocellulose filter membranes (Millipore, IPVH00010). Membranes were incubated overnight at 4℃ with primary antibodies, followed by secondary antibodies incubated 1.5 h at room temperature. Chemiluminescent signals were detected using a CCD camera in a ChemiDoc XRS instrument (Bio-Rad, USA) with Image Lab software.

### Immunohistochemistry (IHC) staining

IHC staining was performed on paraffin-embedded tissue sections as previously described.^25^ Tissues were incubated overnight at 4℃ with ACAA1 antibody (1:200, Abcam, ab90647), followed by anti-rabbit-HRP at room temperature for 30 min. The 3,3-diaminobenzidine (DAB, ZSGB-BIO, ZLI−9018) was used for visualisation, and hematoxylin counterstaining was performed. Two pathologists independently scored the intensity of ACAA1 staining.^27^

### Immunofluorescence staining

Immunofluorescence staining was performed as previously described.^28^ In brief, cells were fixed with 4% formaldehyde for 15 min, permeabilized with 0.5% Triton X−100 for 10 min, and blocked with 5% BSA for 30 min. Primary antibodies were incubated overnight at 4℃, followed by secondary antibody at room temperature for 1 hour. Cells were double stained with rhodamine phalloidin for 30 min at room temperature and DAPI for nuclear visualisation. Immunofluorescence images were obtained using a confocal microscope (Olympus, FV3000) and analysed by ImageJ software.

### CCK−8 viable cell counting assay

Cells were seeded in 96-well plates (2 × 10^3^ cells/well) and incubated for five days. CCK−8 solution (10 μl) (Dojindo Laboratories, CK04) was added and incubated at 37℃ for 2 hours in the dark. Absorbance at 450 nm was measured. Experiments were performed in triplicate.

### Colony formation assay

Cells were seeded in six-well plates (100 cells/well), and cultured for 14 days. Colonies were stained with Giemsa, photographed, and counted using Quantity One 1-D Analysis Software (v4.4.0). Experiments were performed in triplicate.

### Wound healing assay

Cells were seeded in six-well plates (2 × 10^5^ cells/well), and cultured in FBS-free DMEM medium. When cells reached a monolayer and confluent state, cells were scratched using 20 μl sterilised pipette tips. Images were captured at 0 and 24 hours with Olympus CKX−41 inverted microscope (100 × magnification). The width of scratch was measured with Image J software. Experiments were performed in triplicate.

### Transwell invasion assay

Cell suspensions (2.5 × 10^4^ cells) in FBS-free DMEM medium were added to the upper chamber of BioCoat Matrigel plates (BD, 354480). DMEM medium with 10% FBS as a chemoattractant was added to the lower chamber of the BioCoat Matrigel plates. After 48 hours, invading cells were fixed, then stained with crystal violet (1%), and photographed with Olympus CKX−41 inverted microscope (100 × magnification). Experiments were performed in triplicate.

### Tumor formation assay *in vivo*

Six-week-old female BALB/c athymic nude mice were purchased from the Experimental Animal Centre of Guangxi Medical University (Nanning, China). Stable transfected CNE1-ACAA1 cells (1 × 10^6^ cells) were injected subcutaneously into the right flank of nude mice. An equal number of CNE1-control cells was injected into the left flank of mice as a control. Tumour growth was monitored for two weeks. On day 14, the mice were euthanized, and tumours were removed and assessed. All procedures were approved by the Committee on the Ethics of Animal Experiments at Guangxi Medical University.

### GO and KEGG enrichment analysis

To explore the potential biological functions and possible pathways involved in ACAA1, Gene Ontology (GO) and Kyoto Encyclopaedia of Genes and Genomes (KEGG) enrichment analyses were conducted via the cluster Profiler packages in R,^29^ with a False discovery rate (FDR) < 0.05 considered statistically significant.

### Correlation of ACAA1 expression with immune cell infiltration

Immune infiltration and tumour microenvironment analysis were performed using IOBR package in R.^30^ NPC patients were divided into ACAA1-high and -low expressing groups based on the median ACAA1 expression (GSE102349). The xCell signature was applied to evaluate the levels of 64 types of immune and stromal cells infiltration, including extracellular matrix cells, epithelia cells, hematopoietic progenitors, innate and adaptive immune cells.^31^ Malignant Tumours using the Expression Data (ESTIMATE) analysis was applied to evaluate tumour purity, stromal and immune cells content.^32^ Immunophenoscore was applied to evaluate the role of ACAA1 in predicting the immunotherapy response in NPC patients.^33^ Further calculation was performed to reveal the correlation between the expression of ACAA1 and 16 immune checkpoint-related genes (PDCD1, CD274, CTLA4, CD80, CD86, LAG3, LMTK3, TIGIT, BTLA, CD40, CD27, CD28, CD47, SIRPA, IDO1, and IDO2).

### BODIPY cell lipid droplet staining

Cells were seeded in 6-well plates and fixed with 4% paraformaldehyde for 15 min after adherence. Bodipy 493/503 (Thermo Fisher, D3922) diluted in 1 × PBS was added and incubated in the dark at room temperature for 30 min. After washing, DAPI (Thermo Fisher, R37606) was used for staining in the dark for 5 min. Anti-quenching mounting medium was then applied and the cells were visualised under a fluorescence microscope.

### Thiobarbituric Acid Reactants (TBARS) fluorometric assay

TBARS levels were measured using TBARS Fluorometric Assay Kit (Elabscience, E-BC-K298-F) in cell samples. Cells were homogenised in 1 × PBS, centrifuged at 10,000 × g for 10 min at 4℃. Standards (0.1mL) and supernatant from the centrifuged test samples were added, sealed, mixed, and incubated at 100℃ for 1 hour. Post-cooling and centrifugation at 1600 × g, supernatant (0.25 mL) was analysed for fluorescence at 520 nm excitation and 550 nm emission. Experiments were performed in triplicate.

### Statistical analysis

All statistical analyses were conducted using the R software (R Foundations for Statistical Computing, version 4.2.1). The ComBat function of the sva package in R was used to remove the batch effect of the merged dataset. The Wilcoxon test was used for two-groups comparisons, while the Kruskal-Wallis test was used for multiple group comparisons. Spearman’s correlation was used to evaluate associations by the Performance Analytics package in R. The prognostic value of ACAA1 for NPC was evaluated and plotted by the time ROC package in R.^34^ The Kaplan-Meier survival analysis was carried out by the survival^35^ and survminer packages in R, with the ideal cutoff point. Statistical significance was set at *p* < 0.05.

## Results

### Downregulation of ACAA1 was negatively correlated with advanced clinical stages of NPC patients

**Figure 1.**
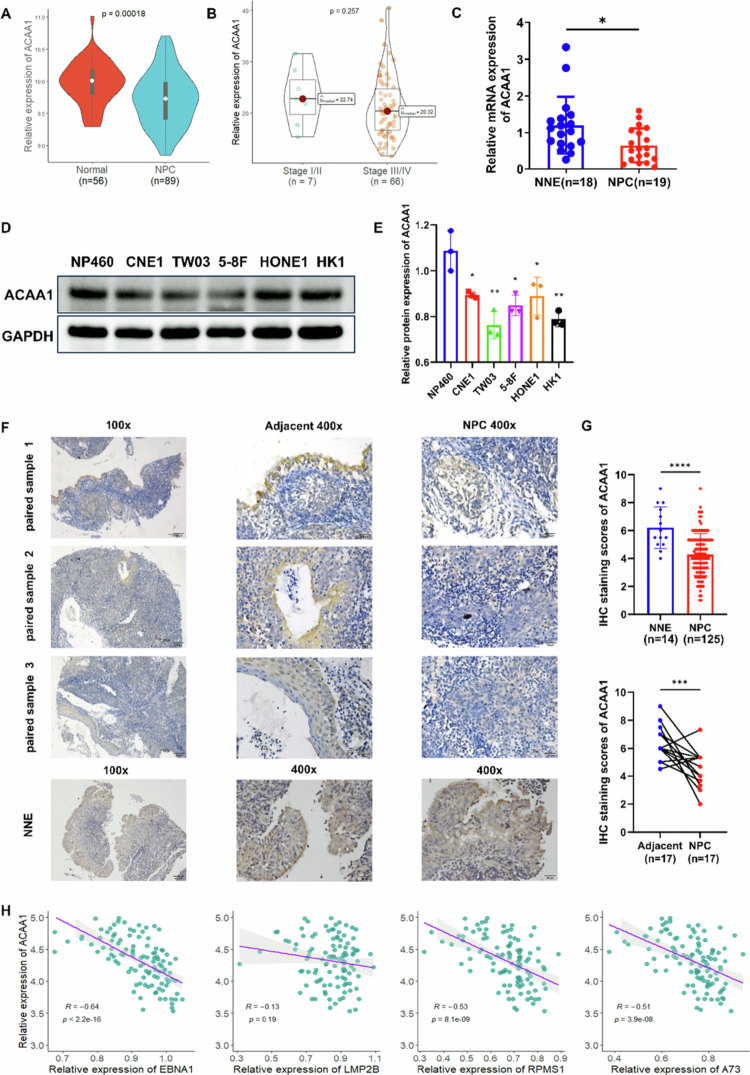
Downregulation of ACAA1 in NPC primary tissues and cell lines. (A) Relative mRNA expression of ACAA1 was analysed using GEO datasets. (B) Differential expression of ACAA1 was analysed between NP C cases in stage I/II and stage III/IV within the GSE102349 dataset. (C) qPCR analysis of ACAA1 mRNA levels in primary NPC tumours (*n* = 19) and NNE tissues (*n* = 18). Data are presented as scatter plots with the 25 to 75 percentiles, and the horizontal line indicates the mean. (D-E) Western blot analysis of ACAA1 protein expression in five NPC cell lines (CNE1, TW03, 5-8F, HONE1, and HK1) and one non-cancerous nasopharyngeal epithelial cell line NP460. GAPDH was used as an internal control. Data are presented as the mean ± SD (*n* = 3). (F-G) IHC staining of ACAA1 in a tissue microarray of 125 primary NPC tissues, 17 paired normal tissues and 14 NNE tissues. Representative images and IHC scores were shown. (H) Correlation between ACAA1 expression and EBV-encoded genes expression. Magnification: × 100, × 400. *P < 0.05, **P < 0.01, ****P < 0.0001.

To investigate the loss of ACAA1 in NPC, four additional independent GEO datasets (GSE180272, GSE53819, GSE61218, and GSE64634) were reassessed. As shown in Figure 1A, Meta-analysis of these datasets revealed significant downregulation of ACAA1 in NPC tissues compared to normal controls (NPC = 89, normal controls = 56, *p* < 0.05). To assess the clinical relevance of ACAA1 expression, we analysed the GSE102349 dataset containing clinical stage information. While a trend toward decreased ACAA1 mRNA levels was observed in advanced-stage (III/IV) NPC patients, this association did not reach statistical significance (Figure 1B, *p* > 0.05), suggesting that ACAA1 downregulation is a persistent event in NPC pathogenesis.

Next, quantitative RT-PCR was carried out in 19 NPC samples and 18 normal nasopharyngeal epitheliums (NNE). Consistent with previous findings, ACAA1 mRNA expression level was significantly diminished in NPC tissues (Figure 1C, *p* < 0.05). This downregulation was consistently observed at the protein level across five NPC cell lines (CNE1, TW03, 5-8F, HONE1, and HK1) relative to the non-malignant nasopharyngeal epithelial cell line NP460 (Figure 1D, E). Additionally, IHC staining revealed that the average ACAA1 staining score was significantly lower in NPC tissues (average staining score:4.28, *n* = 125) when compared to NNE (average staining score: 6.21, *n* = 14) (Figure 1F and G, *p* < 0.0001). Notably, there were 17 cases in which matched tumour and adjacent normal tissues were available, significantly consistent reduction of ACAA1 expression in tumour tissues was observed, reinforcing the tumour-specific nature of ACAA1 downregulation in NPC.

EBV is a well-known aetiological factor in NPC, plays a crucial role in both tumour initiation and progression through its encoded oncoproteins. Given that EBV is closely linked to NPC pathogenesis, we further explored the relationship between ACAA1 and EBV-related factors. To elucidate potential interplay between ACAA1 and EBV-mediated oncogenesis, we analysed their correlation. As shown in Figure 1H, ACAA1 expression showed a significant negative correlation with EBNA1, RPMS1, and A73 (correlation coefficients of −0.64, −0.53, and −0.51, respectively; all *p* < 0.05). While the correlation between ACAA1 and LMP2B was not statistically significant (*p* > 0.05), a trend toward a negative association was observed. ACAA1 expression was negatively correlated with EBV-encoded genes.

### ACAA1 is an effective diagnostic and prognostic biomarker of NPC

**Figure 2.**
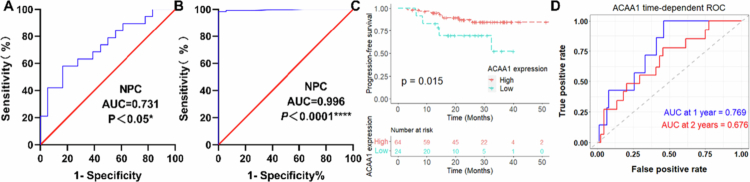
ACAA1 as a potential biomarker for diagnosis and prognosis of NPC. ROC curves were used to evaluated the diagnostic efficacy of ACAA1 expression levels based on qRT‐PCR (A) and IHC scores (B). (C) Kaplan-Mier plot showing the difference in progression-free survival (PFS) between high and low ACAA1 expressing NPC groups. (D) Time-dependent ROC curves for ACAA1 at 1-year and 2-year PFS.

Our diagnostic analysis revealed that ACAA1 mRNA expression levels effectively discriminate between NPC and normal controls, with an area under the curve (AUC) of 0.771 (Figure 2A, *p* < 0.01). Notably, the AUC for ACAA1 protein expression based on IHC scores, was higher at 0.996 (Figure 2B, *p* < 0.0001), suggesting that ACAA1 expression, at both mRNA and protein levels, represent a highly promising biomarker for NPC detection.

To assess the prognostic significance of ACAA1, we stratified NPC patients from the GSE102349 cohort into high and low expression groups based on median ACAA1 expression levels. Kaplan-Meier survival analysis demonstrated significantly longer progression-free survival (PFS) in patients with high ACAA1 expression compared to those with low expression (Figure 2C, *p* < 0.05), supported by a Harrell's concordance index of 0.633 (95% CI: 0.510−0.757). The prognostic utility of ACAA1 was further validated through time-dependent ROC analysis, which yielded AUC values of 0.769 and 0.676 for 1-year and 2-year survival predictions, respectively (Figure 2D). Consequently, reduced ACAA1 expression is associated with an unfavourable prognosis, underscoring its critical role in the NPC progression and clinical outcomes.

### Exogenous ACAA1 expression inhibits proliferation, migration, and invasion of NPC cells

**Figure 3.**
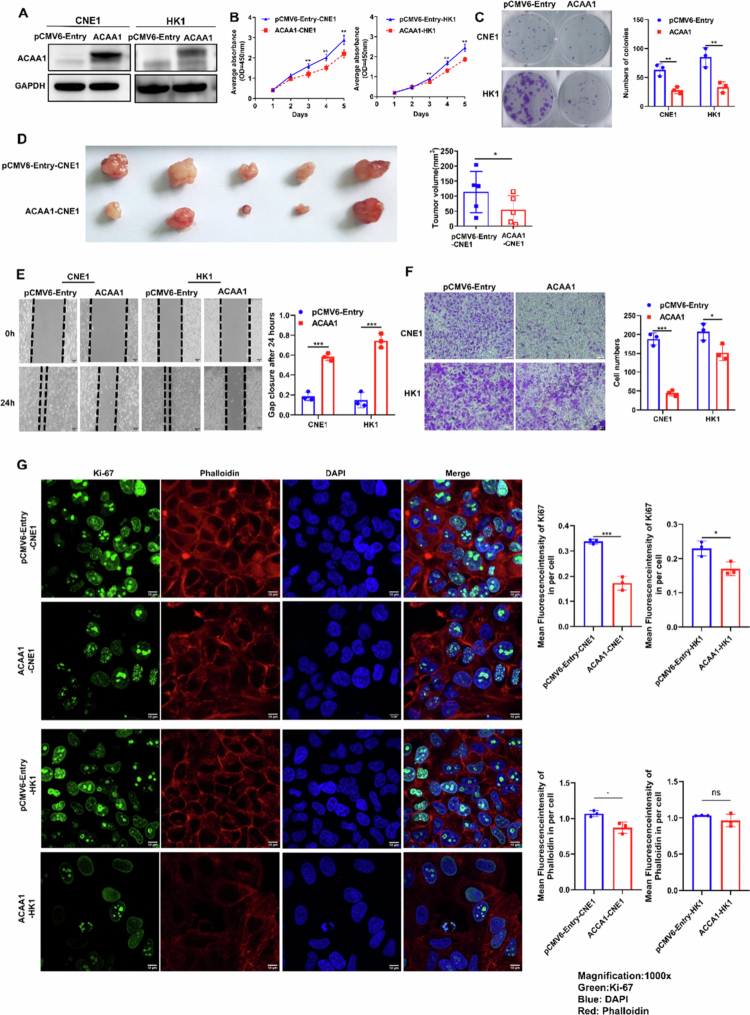
Exogenous expression of ACAA1 suppresses the cell proliferation, migration, and invasion of NPC cells. (A) Western blot analysis confirming the ACAA1 expression in CNE1 and HK1 cells stably transfected with ACAA1-pCMV6-Entry or pCMV6-Entry vector. (B) CCK8 assay assessing cell proliferation in ACAA1-CNE1/HK1 and pCMV6-Entry-CNE1/HK1 cells. Data are presented as the mean ± SD (*n* = 5). (C) Colony formation assay showing the colony number in ACAA1-CNE1/HK1 and pCMV6-Entry-CNE1/HK1 cells. Data are presented as the mean ± SD (*n* = 3). Representative images are shown. (D) Xenograft tumours were removed from nude mice 14 days post-inoculation. The bar chart showed the average volume. Data are presented as the mean ± SD (*n* = 5). (E) Wound healing assay measuring cell migration in ACAA1-CNE1/HK1 and pCMV6-Entry-CNE1/HK1 cells. Images were taken at 0 and 24 hours, and the gap closures rate was measured as [(width at 24 h)/(width at 0 h)]. (F) Transwell assay measuring the invasion ability of ACAA1-CNE1/HK1 and pCMV6-Entry-CNE1/HK1 cells. Invading cells were stained with crystal violet and counted. Representative images were shown. Data are presented as the mean ± SD (*n* = 3). (G) Immunofluorescence staining for Ki−67 and phalloidin to assess cell proliferation and cytoskeletal structure. The mean grey value of Ki−67 and phalloidin per cells was shown in bar chart. Green: Ki−67; Red: phalloidin; Blue: DAPI. Magnification: × 1000. *P < 0.05, **P < 0.01, ***P < 0.001.

To assess the impact of ACAA1 on NPC cells, we established stable ACAA1-overexpressing cell lines ACAA1-CNE1/HK1 and corresponding control cell lines pCMV6-Entry-CNE1/HK1. Western blot analysis confirmed successful ACAA1 overexpression (Figure 3A). CCK8 assays exhibited significantly slower growth in ACAA1-CNE1/HK1 cells compared to controls (Figure 3B, *p* < 0.01). In colony formation assays, ACAA1- overexpressing cells formed fewer and smaller colonies than controls (Figure 3C, *p* < 0.01). Additionally, immunofluorescence staining revealed a significant reduction in Ki−67 expression in ACAA1-CNE1/HK1 cells (Figure 3G; ACAA1-CNE1: *p <* 0.001, ACAA1-HK1 *p <* 0.05). These results further indicate that ACAA1 suppresses proliferative capability of NPC cells.

To validate these findings *in vivo*, ACAA1-CNE1 cells and control cells were injected into the flanks of nude mice, respectively. While all nude mice developed tumour masses, those derived from ACAA1-CNE1 cells were significantly smaller than those from control cells (Figure 3D, *p* < 0.05), indicating that ACAA1 overexpression inhibits tumour growth in NPC *in vivo*.

To assess the impact of ACAA1 on cell motility, we performed wound healing assays, which revealed delayed wound closure in ACAA1-CNE1/HK1 cells (Figure 3E, *p <* 0.001). Additionally, transwell assays demonstrated reduced invasive potential in ACAA1-CNE1/HK1 cells after 48 hours (Figure 3F; ACAA1-CNE1: *p <* 0.001, ACAA1-HK1 *p <* 0.05).

To visualise microfilament morphology, phalloidin staining was performed. A significant reduction in F-actin levels was observed in ACAA1-CNE1 cells, with fluorescence predominantly accumulated at the leading edges (Figure 3G; ACAA1-CNE1: *p*＜0.05, ACAA1-HK1 ns). We thus proposed that ACAA1 overexpression might impair NPC cells migration by disrupting the intracellular cytoskeleton through depolymerisation and redistribution of F-actin.

In conclusion, exogenous ACAA1 expression inhibits the proliferation, migration, and invasion of NPC cells, highlighting its potential as a novel tumour suppressor.

### ACAA1 mitigated oxidative stress via inhibiting lipid droplet accumulation in NPC

**Figure 4.**
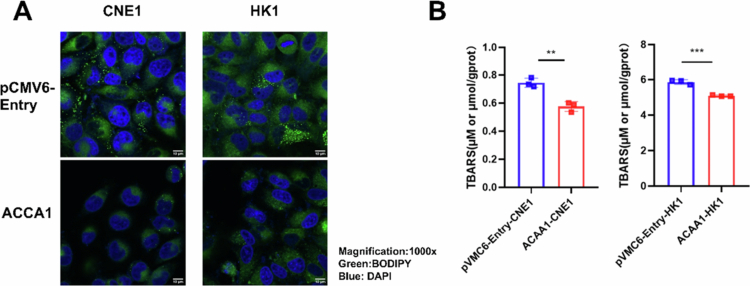
ACAA1 may mitigate oxidative stress by influencing lipid droplet accumulation. (A) Lipid droplet accumulation in CNE1 and HK1 cells transfected with ACAA1-pCMV6-Entry or pCMV6-Entry, analysed via BODIPY staining. Representative images were shown. (B) TBARS assay to assess oxidative stress levels in ACAA1-CNE1/HK1 and pCMV6-Entry-CNE1/HK1 cells. The TBARS concentration was calculated using the formula: TBARS (μmol/gprot) = (∆F-b)/a × f/Cpr. a: the slope of the standard curve; b: the intercept of the standard curve; ΔF: the absolute fluorescence value, (sample fluorescence/blank fluorescence); f: the dilution factor; Cpr​: the protein concentration (gprot/L). *P < 0.05, **P < 0.01, ***P < 0.001.

Due to its role in metabolic regulation, we investigated the effect of ACAA1 on changes in lipid accumulation in NPC cells. Figure 4A showed a reduction in lipid droplets numbers in ACAA1-CNE1/HK1 cells. Additionally, ACAA1 overexpression decreases TBARS levels (Figure 4B; ACAA1-CNE1: *p <* 0.01, ACAA1-HK1 *p <* 0.001), indicating a reduction in lipid peroxidation and oxidative stress. These results suggest that ACAA1 may mitigate oxidative stress by influencing lipid droplet accumulation in NPC cells.

### ACAA1 exerts a positive regulatory effect on immune cell infiltration and the tumour immune environment in NPC

**Figure 5.**
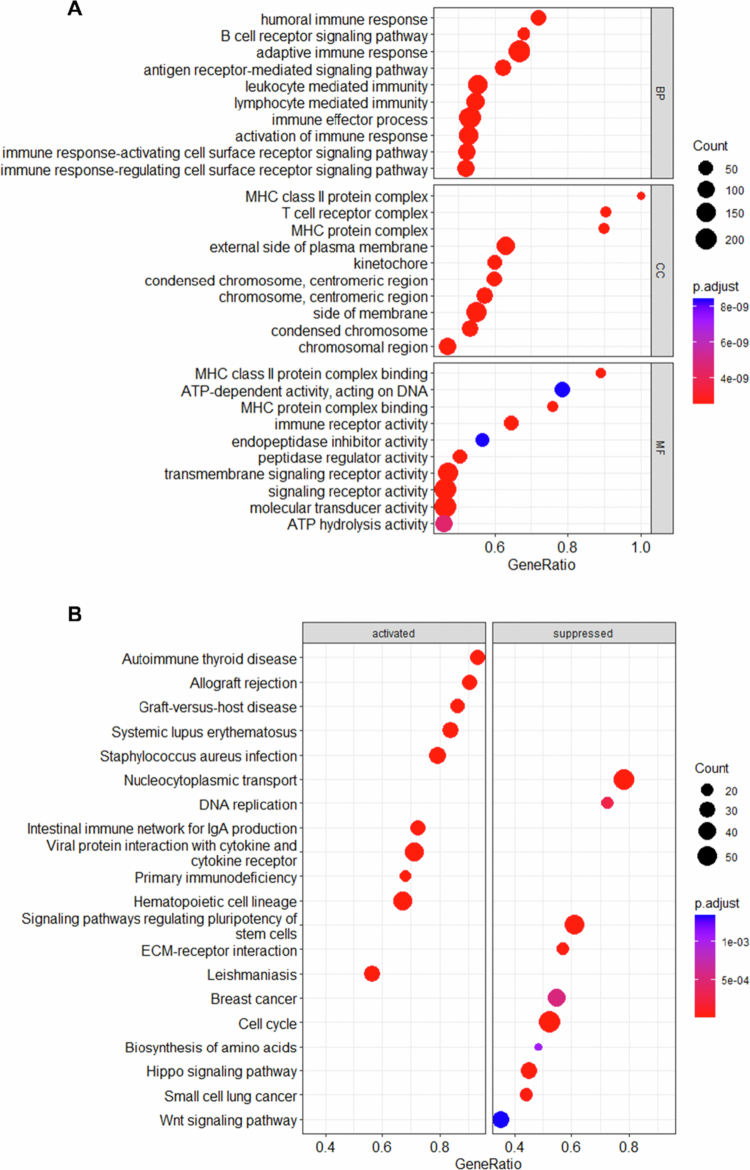
GO and KEGG enrichment analysis. (A) GO enrichment analysis. (B) KEGG pathway enrichment analysis. GO: Gene Ontology; KEGG: Kyoto Encyclopaedia of Genes and Genomes; BP: biological process; CC: cellular component; MF: molecular function.

To uncover the potential biological functions and pathways influenced by ACAA1, we conducted GO and KEGG enrichment analyses using the GSE102349 dataset. GO analysis revealed that ACAA1 was strongly associated with the immune-related terms, including leucocyte mediated immunity, lymphocyte-mediated immunity, immune effector process, adaptive immune response, and immune response-activating/regulating cell surface receptor signalling pathway. (Figure 5A). KEGG analysis showed that high ACAA1 expression correlates with signalling pathways such as allograft rejection, graft-versus-host disease, staphylococcus aureus infection, and viral protein interaction. Conversely, low ACAA1 expression was linked to pathways related to pluripotency of stem cells, cell cycle, hippo signalling pathway, nucleocytoplasmic transport, and Wnt signalling pathways (Figure 5B). Thus, it is hypothesised that ACAA1 plays a role in regulating immune response in NPC cells.

**Figure 6.**
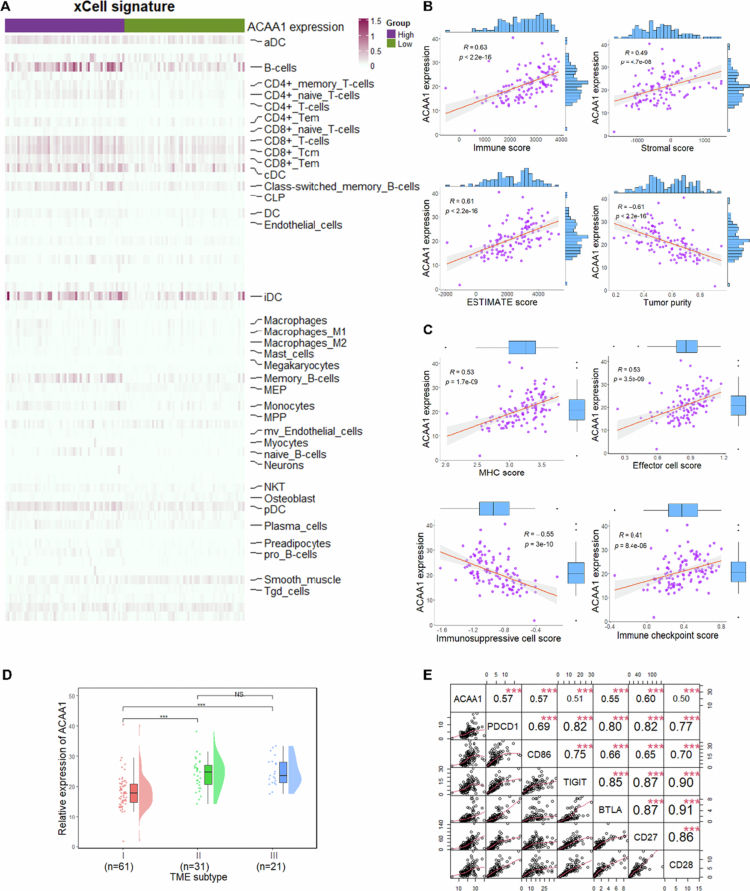
The potential role of ACAA1 in NPC immune microenvironment and immunotherapy. (A) Infiltration levels of 64 immune cell subtypes in high and low ACAA1 expressing groups. Statistically significant subtypes were shown (*P* < 0.05). (B) ESTIMATE analysis of the correlation between ACAA1 expression and immune score, stromal score, ESTIMATE score and tumour purity. (C) Immunophenoscore analysis of the association between ACAA1 expression and MHC score, effector cell score, immunosuppressive score, and immune checkpoint score. (D) Correlation between ACAA1 expression and TME-based subtypes. (E) Correlation analysis between the expression of ACAA1 and six immune checkpoint-related genes (correlation coefficient ≥ 0.5). *P < 0.05, **P < 0.01, ***P < 0.001; NS, no statistical difference.

Next, we further investigated correlation between ACAA1 expression and immune cells infiltration using GSE102349 dataset on the xCell platform. Our analysis revealed a notable elevation in the infiltration of several immune cell types, including activated dendritic cells (aDCs), B cells, CD4 + T cells (including memory T cells, naïve T cells, and Tem), CD8 + T cells (encompassing naïve T cells, Tcm, and Tem), classical DCs (cDCs), macrophages, and both M1 and M2 macrophages in ACAA1-high expressing NPC patients (Figure 6A).

Through ESTIMATE analysis, we found that ACAA1 expression positively correlated with immune score (*p* < 0.001, R = 0.63), stromal score (*p* < 0.001, R = 0.49), and ESTIMATE score (*p* < 0.001, R = 0.61), each displaying significant associations. while negatively correlated with tumour purity (*p* < 0.001, R = −0.61) (Figure 6B).

Further analysis using Immunophenoscore (IPS) revealed significant positive correlations between ACAA1 and MHC score (*p* < 0.001, R = 0.53), effector cell score (comprising activated CD8+ T cells, CD4+ cells, Tem CD8+ cells, and Tem CD4+ cells) (*p* < 0.001, R = 0.53), and immune checkpoint score (*p* < 0.001, R = 0.41). Conversely, ACAA1 expression exhibited a negative correlation with the immunosuppressive cell score (Tregs and MDSCs) (*p* < 0.001, R = −0.55). These results suggest that ACAA1 may play a key role in immune modulation in NPC cells, potentially evading immune surveillance by suppressing its expression (Figure 6C).

### ACAA1 might act as a potential immune-related prognostic and immune-therapeutic biomarker in NPC

The GSE102349 dataset classifies NPC into three subtypes based on immune and stromal genes expression: TME Ⅰ, Ⅱ, and Ⅲ.^36^ Our analysis showed that ACAA1 expression was statistically lower in TME subtype I compared to subtype II and III (TME subtype Ⅰ vs. subtype Ⅱ, *p* < 0.001; TME subtype Ⅰ vs. subtype Ⅲ, *p* < 0.001), suggesting that reduced ACAA1 expression may be associated with poor clinical outcomes in NPC (Figure 6D). This finding aligns with K-M analysis, supporting ACAA1 as a potential immune-related prognostic biomarker.

To evaluate the potential of ACAA1 as a biomarker for immunotherapy, we analysed its correlation with six immune checkpoint-related genes. A significant positive correlation was found between ACAA1 expression and CD27, PDCD1, CD86, BTLA, TIGIT, and CD28 (Figure 6E, all *p* < 0.05). Based on these associations, we hypothesise that high ACAA1 expression may indicate a tumour immune microenvironment more receptive to immune checkpoint blockade, positioning ACAA1 as a promising predictive biomarker for patient stratification.

## Discussion

Emerging evidence has established ACAA1 as a tumour suppressor across multiple malignancies, with its under-expression consistently correlating with unfavourable clinical outcomes. Reduced ACAA1 expression has been linked to enhanced tumour progression and poorer survival in cancers including hepatocellular carcinoma,^37^ breast cancer,^38^ and non-small cell lung cancer.^24^ The tumour-suppressive role of ACAA1 is further supported by genetic evidence: the single nucleotide polymorphism (SNP) rs4988453, located in the promoter region shared by ACAA1 and MYD88 (a downstream effector of toll-like receptors), has been shown to decrease transcriptional activity of ACAA1 and correlate with reduced survival in colorectal cancer patients.^39^ Mechanistically, studies have revealed that ACAA1 expression can be suppressed by oncogenic KRAS through the MAPK signalling pathway.^24^ Aligned with these findings, our study confirms that ACAA1 downregulation is a prevalent feature in NPC. ROC analysis demonstrated that ACAA1 exhibits significant diagnostic potential for NPC at both mRNA and protein levels, warranting further validation in larger clinical cohorts. Furthermore, our functional studies revealed that ACAA1 overexpression effectively suppresses malignant behaviours in NPC cells. Preliminary mechanistic insights suggest that this tumour-suppressive effect may involve ACAA1-mediated modulation of actin dynamics, including inhibition and spatial reorganisation of actin filaments. Together, these results highlight ACAA1 as a promising diagnostic marker and therapeutic target in NPC.

EBV infection is a major aetiological factor in NPC, driving metabolic reprogramming and immune evasion through various encoded genes and signalling pathways.^40–43^ In our study, we observed a significant negative correlation between ACAA1 expression and EBV-encoded genes (EBNA1, RPMS1, and A73), implying a potential interaction between ACAA1 downregulation and latent EBV infection. Further studies are needed to elucidate the mechanistic basis of this relationship.

Previously, we demonstrated that NPC tumour tissues and cell lines show significantly increased LD accumulation.^26^ As a key regulator of lipid metabolism, ACAA1 catalyses the *β*-oxidation of very long fatty acids in the final step of the PPAR signalling pathway. Reduced expression of ACAA1 may impair fatty acid *β*-oxidation, resulting in abnormal lipid accumulation in NPC cells. Conversely, ACAA1 overexpression enhances fatty acid *β*-oxidation and decreases LD accumulation, underscoring its essential role in modulating lipid metabolism in NPC, and consequently influencing tumour cell survival and proliferation. Lipid peroxidation constitutes a central event in ferroptosis. Intracellular enzymes such as lipoxygenases catalyse the peroxidation of polyunsaturated fatty acids, generating lipid hydroperoxides and reactive end-products including 4-hydroxynonenal and malondialdehyde. These compounds disrupt cellular membrane integrity, ultimately leading to loss of cell viability and ferroptosis. Glutathione peroxidase 4 serves as a key antioxidant enzyme that counteracts lipid peroxidation by utilising glutathione to reduce lipid hydroperoxides to non-toxic alcohols. Inhibition of GPX4 activity or depletion of GSH results in unchecked peroxidation and induction of ferroptosis.^44^ Supporting this mechanism, a study in ccRCC had shown that MLYCD-mediated fatty acid oxidation disrupts the homoeostasis of the endoplasmic reticulum and mitochondria, elevates ROS production, and induces ferroptosis, thereby inhibiting ccRCC progression.^45^ Given these findings, activating ACAA1-mediated fatty acid *β*-oxidation may represent a promising therapeutic strategy for NPC.

Fatty acid metabolism is closely linked to immune regulation. Metabolites such as long-chain fatty acids (LCFAs) and polyunsaturated fatty acids (PUFAs) play crucial roles in modulating the metabolism and function of immune cells. For instance, CD8 + T cells rely on fatty acid metabolism to sustain their survival and effector functions within the tumour microenvironment.^46^ Furthermore, dysregulated fatty acid metabolism can influence immune cell polarisation, thereby potentially facilitating tumour immune escape.^47^^,^^48^ ACAA1 has emerged as a significant player in immune-related pathological processes and cancer treatment responses. Genetic evidence highlights its immuno-modulatory potential, as demonstrated by the ACAA1 SNP (rs156265) that modifies endotoxin-induced childhood asthma risk.^49^ In the lung tumour microenvironment, ACAA1 exhibits a strong positive correlation with enhanced antigen presentation and increased infiltration of diverse T cell populations, including CD4 + T cells, Th1, Th2, and Treg subsets.^23^^,^^24^ Clinically, elevated ACAA1 levels have been correlated with improved responses to targeted therapies such as the EGFR inhibitor Erlotinib and VEGFR2/3 inhibitor ZD−6474.^23^ This is particularly relevant for NPC treatment, as Erlotinib has been shown to potentiate radiotherapy efficacy by inducing G2/M phase cell cycle arrest and enhance chemoradiotherapy outcomes through inhibition of DNA damage repair mechanisms.^50^ Our findings corroborate and extend these observations, demonstrating that high ACAA1 expression in NPC is associated with enrichment of immune-related pathways, increased immune cell infiltration, and elevated expression of immune checkpoint-related genes. In addition, the infiltration of immune cells in TME plays a critical role in tumour promotion and progression.^51^^,^^52^ Our data indicate that ACAA1 expression is negatively correlated with immunosuppressive cell populations and positively correlated with effector immune cells, supporting a role for ACAA1 in reshaping the immune landscape and mitigating immunosuppression in NPC. Based on these findings, we propose a model in which ACAA1 modulates immune cell infiltration within the tumour microenvironment, thereby influencing NPC progression and survival. These insights position ACAA1 as a potential therapeutic target for reprogramming anti-tumour immunity in NPC.

Current immunotherapeutic approaches for cancer include immune checkpoint blockade, adoptive cell transfer, and therapeutic vaccination. Immune checkpoint inhibitors (ICIs), which target inhibitory receptors such as PD−1, CTLA−4, and LAG−3, aim to reinvigorate antitumor T cell responses and have shown encouraging activity in NPC.^53–56^ Combination therapies involving PD−1 inhibitors and conventional chemotherapy have yielded superior clinical outcomes in terms of both overall survival (OS) and progression-free survival (PFS).^57^^,^^58^ The field continues to advance with the development of dual checkpoint inhibitors targeting pairs such as CTLA−4/PD-L1, LAG−3/PD-L1, and TIM−3/PD-L1, which are under clinical evaluation.^59^ Nonetheless, the utility of these agents remains constrained by regulatory and intrinsic limitations of monotherapy. Our study adds to this landscape by revealing a positive correlation between ACAA1 expression and key immune checkpoint genes (e.g., PDCD1, CTLA4, CD80, CD86, LAG3), suggesting that ACAA1 may serve as a prospective biomarker for identifying NPC patients who could benefit from immunotherapy, thus aiding in patient stratification.

In conclusion, ACAA1 acts as a pivotal tumour suppressor in NPC through integrated effects on lipid metabolism, oxidative stress, cytoskeletal organisation, and immune modulation. Its expression level may serve as a prognostic biomarker and a predictor of immunotherapy response. These insights not only deepen our understanding of NPC pathogenesis but also propose ACAA1 as a candidate biomarker for metabolic for guiding treatment.

## Data Availability

The datasets used and/or analysed during the current study are available from the corresponding author on reasonable request.
